# The effects of using an auto-subtitle system in educational videos to facilitate learning for secondary school students: learning comprehension, cognitive load, and satisfaction

**DOI:** 10.1186/s40561-023-00224-2

**Published:** 2023-01-10

**Authors:** Sivakorn Malakul, Innwoo Park

**Affiliations:** Department of Education, College of Education, Korea University, 145 Anam-ro, Seongbuk-gu, Seoul, 02841 Korea

**Keywords:** Auto-subtitles, Subtitles, Educational videos, Learning comprehension, Cognitive load, Satisfaction

## Abstract

While subtitles are considered a primary learning support tool for people who cannot understand video narration in foreign languages, recent advancements in artificial intelligence (AI) technologies have played a pivotal role in automatic subtitling on online video platforms such as YouTube. This study examines the effects of three different types of subtitles in the Thai language (i.e., auto-subtitles, edited subtitles, and no subtitles) on learning comprehension, cognitive load, and satisfaction to determine whether it is feasible to use AI technology as an auto-subtitles system to facilitate online learning with educational videos. To that aim, 79 Thai secondary school students from three Mathayom 5 (Grade 11) computer science classrooms participated in this study. This study used the static group comparison, which is the Posttest-Only Control Group Design. The results of this study found that the auto-subtitles system that generates Thai language subtitles for English educational videos has greater feasibility of implementation to facilitate online learning when compared to editorial subtitles by Thai natives. Therefore, Thai subtitles generated by the auto-subtitles system in English educational videos can facilitate students’ learning comprehension, cognitive load, and satisfaction.

## Introduction

In this digital age of learning, massive open online courses (MOOCs) provide learners with global access to a wide range of available learning content. In particular, most video content on platforms is instructed in English or their native language to serve their primary audience in the local country (Cho & Byun, 2017). Therefore, when applying those video content in educational settings in countries that use different languages, accessibility can matter.

Due to a language barrier, students might have difficulties understanding content, eventually excluding some of them and becoming less proficient in English. For example, when non-English speakers view video content in the English language without subtitles, their cognitive load is higher than when using subtitles (Liao et al., 2020), and they have less learning motivation (Kruger et al., 2017). Likewise, according to Nonthamand (2017), language proficiency was the biggest obstacle for Thai students when learning video content on a MOOC platform. To overcome these difficulties, some educators have created courses containing video lectures with captions to support local language learners. However, creating multi-language subtitles in foreign languages has not yet become a specific requirement for online lesson production.

To alleviate this problem, subtitling and captioning can be considered. Theories such as the cognitive theory of multimedia learning (Mayer, 2014) and the bilingual dual coding theory (Paivio, 1990, 2014; Paivio & Desrochers, 1980) support this claim. Mayer’s cognitive theory of multimedia learning suggests that combining words and images has a more significant learning impact when people receive information than just words alone (Mayer, 2014). The advantages of using captions and subtitles in videos to facilitate learning for both native and non-native speakers are shown in several different studies (Cao et al., 2019; Danan, 2004; Garza, 1991; Guillory, 1998; Hsieh, 2019; Liao et al., 2020; Liyanagunawardena, 2021; Shadiev et al., 2015; van der Zee et al., 2017). Captioning a video helps learners visualise what they hear (Danan, 2004), bridges the gap between their listening and reading (Garza, 1991), and allows them to clarify any unclear accents or parts of the video with low audio quality (Liyanagunawardena, 2021). Furthermore, captions and subtitles make learners feel comfortable because they can readily access meaning (Guillory, 1998) and predict content (van der Zee et al., 2017) as well as increase their immersion with characters that fulfil a focusing effect to gain a stronger connection with the story (Kruger et al., 2017). Nevertheless, making captions and translating subtitles may be considered costly in terms of time and budget. The normal transcription process is expensive and time-consuming, requiring dedicated employees or services and a significant amount of physical work (Dachowski, 2018; Liyanagunawardena, 2021).

Yet, with technological advancement, artificial intelligence (AI) technology enabling auto-subtitling is now available, but the technology’s feasibility is yet to be proven. These advanced technologies consist of systems that drive two parts: Automated Speech Recognition (ASR), also known as speech-to-text, and Neural Machine Translation (NMT). The ASR automatically generates the caption of the speaker’s words in the video, while the NMT simultaneously translates the caption into the target language.

As mentioned above, to overcome obstacles related to learning with educational or instructional videos in foreign languages and expand educational opportunities to make learning more boundless, this study aims to (1) experiment with the feasibility of using AI technology as an auto-subtitles system to facilitate online learning with educational videos. (2) Examine the effects of using the auto-subtitles system in educational videos to facilitate learning comprehension, cognitive load, and satisfaction. The research questions proposed in this study were as follows:

1. Can subtitles generated by an auto-subtitles system facilitate learning comprehension reach the same level as edited subtitles on an educational video?
2. Do auto-subtitles and edited subtitles have different levels of cognitive load?
3. Are students satisfied with using auto-subtitles systems?

## Background

### Learning with captions and subtitles

Although captions and subtitles are both considered virtual-text information (Liyanagunawardena, 2021), they slightly differ based on the purpose of using them. ‘Subtitles’ are a direct transcription or translation to represent the exact dialogue of the conversation as overlaid texts that are suitable for audiences unfamiliar with the spoken language in the media. While ‘captions’ include not only a transcription or translation of spoken dialogue to text elements in the same languages, but they also contain environmental sounds, sound effects, music, and other audio cues for audiences when sound is unavailable or unclear audible, as well as hearing disorders (Cersosimo, 2019; UCL, 2020; WHATWG, 2021). Thus, subtitles are considered in terms of dialogue translation, while captions describe all sounds that happen and insert more information into the media. Additionally, to explain the cognition learning process with captions and subtitles, this study refers to the bilingual dual-coding theory of Paivio (2014), which is commonly applied to learning in foreign language studies.

Bilingual dual-coding theory refers to the extension of the previous “*Dual Coding Theory*” of Allan Paivio (Paivio & Desrochers, 1980). On the one hand, the dual coding theory assumes that humans remember well when receiving information from more than two channels: verbal or auditory information and image or visual information. On the other hand, the key assumptions of the bilingual dual-coding theory are concentrated on people who are bilinguals creating separate recognition processes for interrelated logogen systems for their first (L1) and second (L2) languages, each of which is linked to a nonverbal imagen system (Paivio, 1990, 2014). The connections of L1–L2 occur between translation-equivalent logogens and may be thought of as a subset of verbal associative relationships with a high likelihood of activation in code-switching tasks. Furthermore, in episodic and semantic memory tasks, the imagen system comprises common and language-specific imagens that can moderate L1–L2 performance.

Previous studies of using captioning and subtitles in education mainly experimented with using them to facilitate learning languages, especially in English as a Foreign Language or EFL, to study the effects of using captions or subtitles to enhance students’ language skills and learning comprehension (Alabsi, 2020; Danan, 2004; Garza, 1991; Guillory, 1998; Hsieh, 2019; Li, 2013; Liyanagunawardena, 2021). Moreover, additional studies were conducted to examine the effects of captions and subtitles on cognitive load and attention (Cao et al., 2019; Kruger et al., 2013, 2017; Liao et al., 2020).

### Auto-subtitle systems

The utilisation of AI-powered machine translations has been applied to language learning as a ‘learning tool’ that is used to manage language learning, known as Computer-Assisted Language Learning (CALL), since the 1980s (Kemble & Brierlay, 1991). These auto-subtitles can shed light on the AI technologies developed over the past several decades that blend ASR and NMT, such as Speech-to-speech translation systems or ‘Media Translation’. It contains three separate components: automatic speech recognition to transcribe the source speech as text, machine translation to translate the transcribed text into the target language, and text-to-speech synthesis (TTS) to generate speech to the target language from the translated text (Jia & Weiss, 2019). Thus, auto-subtitles are an automatic system that recognises the speech or voice from L1 and appears as translated visual-text information in the target language on the video. As mentioned in the previous section, subtitles have advantages for learners learning in their L2. Although the current process of making subtitles in the target language has a high cost on staff and time (Dachowski, 2018), auto-subtitles systems could support production, raise consumption, and facilitate learning. For instance, the videos currently being published on online video platforms, such as YouTube, allow the creator to provide captions and translated subtitles by semi-automatic features, which the creator can re-edit with the correct captions or subtitles on their videos. Additionally, audiences can enable auto-captioning and translate the caption by themselves. According to the features on YouTube, the auto caption and auto-translation on the YouTube video player allows learners to access the subtitles freely.

As mentioned above, ASR is the core engine of auto-captioning, which is also considered in this study. In previous studies, the effects of using ASR to improve language learning and learning performance (Kuo et al., 2012; Shadiev et al., 2014, 2015; Wang & Young, 2014), cognitive load (Chan et al., 2020) as well as visual attention and learning behaviour (Huang et al., 2014) have been examined. In addition to ASR, NMT has been studied to enhance performance in learning languages, including speaking and writing comprehension (Briggs, 2018; Lee, 2019; Niño, 2020) and reading comprehension (Fuji, 1999; Castilho & Arenas, 2018). However, there is currently a lack of research considering both technologies together. Therefore, this study intends to investigate the combination of ASR and NMT as an auto-subtitles system to facilitate learning.

The positive outcomes of utilising auto-captions and auto-translation could be synthesised according to the previous studies mentioned above. Both auto-caption and auto-translation have experimented with improving students’ learning, and the advantage of visual-text information and translated information positively facilitating learning is categorised in Table 1

**Table 1:** Positive outcomes of using captions/subtitles and translation in education

Tools	Outcomes
Manual	
Captions	Bridging listening and reading comprehension (Garza, 1991) Listening comprehension (Li, 2013; Liyanagunawardena, 2021) Content prediction (van der Zee et al., 2017) Content concentration (Kruger et al., 2017) Vocabulary and Learning comprehension (Hsieh, 2019) Learning outcome and Cognitive load (Cao et al., 2019)
Subtitles	Content comprehension (Danan, 2004) Listening comprehension (Alabsi, 2020) Learners feel comfortable while watching videos (Guillory, 1998) Cognitive load (Kruger et al., 2013) Immersion (Kruger et al., 2017) Visual attention, Cognitive load, and Comprehension (Liao et al., 2020)
Automatic	
Auto-captions (ASR)	Listening comprehension and Learning concentration (Shadiev et al., 2015) Learning performance (Chan et al., 2020; Kuo et al., 2012) Visual attention (Huang et al., 2014) English pronunciation (Wang & Young, 2014)
Auto-translation (NMT)	Speaking and Writing comprehension and performance (Briggs, 2018; Niño, 2020) Writing performance (Lee, 2019) Reading comprehension (Fuji, 1999; Castilho & Arenas, 2018)

From Table 1, several related variables represent the pros of using captions and subtitles in educational videos. Moreover, using AI in education (i.e., ASR and NMT) can enhance learning. More specifically, the four main outcomes of facilitating learning include learning language skills performance, learning comprehension, learning concentration, learning comfortably, and cognitive load. Nevertheless, due to the experiment’s time constraints, measuring learners’ language skill improvement over a short period may be unrealistic. Plus, the hardware and specific software to measure students’ learning concentration through eye-tracking analysis while learners watch the videos (Huang et al., 2014; Kruger et al., 2017; Liao et al., 2020; Shadiev et al., 2015) was unavailable. Lastly, while it could be possible to investigate the acceptance of using technologies through learner comfort level, this study will examine learners’ satisfaction instead. Consequently, this study intends to use carefully crafted research questions to examine the experiment’s results on learners’ comprehension, cognitive load, and satisfaction.

## Methodology

### Participants

Students who participated in this experiment were 79 students in grade 11 (Mathayom 5) from the secondary demonstration school under the faculty of education of a public university in Bangkok. All of the students studied science and mathematics programmes as well as distributed levels of educational achievement in each classroom. The demographic characteristics of participants in terms of age, gender, and the number of students in each experiment group are shown in Table 2. This study used stratified sampling (Cohen et al., 2007) to assign a single treatment to each of the three classrooms. There were two groups of experimental treatments (i.e., 1 with auto-subtitles and 1 with edited subtitles) and a single control group with no subtitles. Teaching was conducted during regular teaching hours. All participants in this research project acknowledged the guideline of the experiment and filled out a consent form with authorisation from their guardians. According to the experiment constructed on the actual date and time of the class, the experiment was blended into the classroom’s learning activities as Classroom Action Research (CAR). All data collected were highly protected and confidential. The participants’ privacy was maintained by replacing classroom numbers with A–C and applying for student orders instead of student identification numbers.

**Table 2:** Demographic characteristics of participants (N = 79)

Characteristics	Frequency	Percentage
Age		
16	59	74.68
17	20	25.32
Gender		
Female	35	44.30
Male	42	53.17
Others	2	2.53
Number of students per group		
Auto-subtitles	27	34.18
Edited subtitles	26	32.91
No subtitles	26	32.91

### Materials

#### Educational videos and subtitles

The educational videos for this study were selected from YouTube and produced by the TED-Ed project (TED-Ed, 2012). These videos were a collection of short animations with narratives in English to be blended into computing science classrooms as learning materials. All students were allowed to watch each video once by the teacher. Also, this study selected two videos related to the context of the classroom’s learning content in a fundamental of AI, and the length of each video was approximately 4–5 min. The main topics of the two videos are as follows; (1) How does AI learn? (Brownell, 2021), and (2) How computers translate human language (Papachimonas, 2015).

Three kinds of subtitles were used in this experiment: auto-subtitles, edited subtitles, and no subtitles. For the auto-subtitles on the videos, this study used YouTube’s auto-captioning and auto-translation system, which is powered by Google, because English videos can use auto subtitles. Next, the edited subtitles in the Thai language of selected videos were translated by Thai native speakers who were named on the videos. Lastly, all videos could turn the subtitles on–off, and the teacher could choose the type of subtitles through the settings button of the video players.

#### Data collection instruments

Four primary instruments were used to collect data in this study: the English listening comprehension test, the video content-learning comprehension test, the Cognitive load scale, and the Learner satisfaction scale.

*First*, this study’s English listening comprehension test applies the Test of English as a Foreign Language in Institutional Testing Program (TOEFL ITP) in the part of listening comprehension in the second level (high beginning to intermediate). This test used 14 questions of test listening practice sets of TOEFL ITP that were created and published on the official website of the Educational Testing Service (ETS, n.d.). Furthermore, the test was divided into three parts (i.e., short sentences, short conversations, and long conversations & lecture talks). The teacher controlled the audio track, and students could only listen to the audio track one time. At the end of each track, students were asked to select the correct answer from multiple-choice questions found on a Google Form. Each question was valued at 1 point, so the total score was 14. After submitting the test, students were informed of their English listening comprehension scores via Google Form’s automated scoring system known as quiz mode. The Cronbach’s alpha reliability test for this sample’s English listening comprehension test after the experiment was 0.84.

*Second*, the video content-learning comprehension test was based on the content of each video and contained five 4-multiple choice questions per video in the Thai language to assess the videos’ content comprehension. This test asked students to choose the correct answer that best matches the content presented in the video. Each question was worth 1 point and had a total score of 5. After submitting a video content-learning comprehension test, the test’s total score was given via the automated scoring system on quiz mode along with the students’ selected answer and the correct answer.

*Third*, this study’s cognitive load scale was adapted from the work of Moon and Ryu (2020), and the five revised constructs used to measure were as follows: (1) task demanding, (2) mental effort, (3) perceived task difficulty, (4) self-evaluation, and (5) usability. Moreover, according to Moon and Ryu (2020), the instrument’s reliability coefficient of each construct had a Cronbach’s alpha average score greater than 0.7. This original instrument refers to synthesising of *the Subjective Workload Assessment Technique (SWAT)*, *the NASA-Task Load Index (TLX),* and *the Subjective Cognitive Load (SCL)*, as mentioned by Ryu (2011). This study’s cognitive load scale employed a 7-rating Likert scale, and the assessment questions were translated and edited into Thai. Moreover, to help ensure validity, all Thai translations were confirmed by 2 experts in educational technology.

*Fourth*, the learner satisfaction scale was adapted from the Learner Satisfaction Scale from Donkor’s (2011) Assessment of learner acceptance and satisfaction with video-based instructional materials based on *the Technology Acceptance Model (TAM)* that Fred Davis proposed in 1989. Donkor (2011) mentioned that Cronbach’s alpha reliability test for the Learner Satisfaction Scale yielded a value of 0.88. In addition, for this study’s learner satisfaction scale, a 4-rating Likert scale was employed, and the assessment questions were translated and edited into Thai. Once again, the experts were asked to confirm all translations into Thai to maintain validity.

### Experimental procedure

**Fig. 1:**
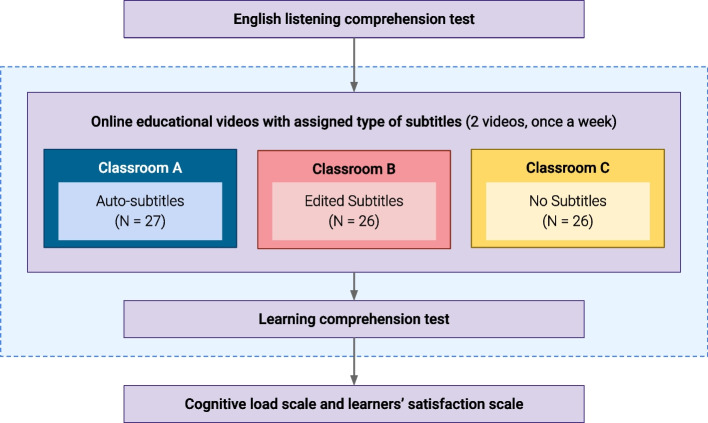
Experimental procedure process flow

According to the experimental research design, this study utilises the static group comparison, which is the Posttest-Only Control Group Design (Morgan & Renbarger, 2018), to test the result between each experimental group (i.e., auto-subtitles and edited subtitles) and the control group (i.e., no subtitle). The experiment was blended in the three computing science classrooms during the second semester of 2021. Throughout the experiment, each classroom was assigned one of the three types of subtitles: auto-subtitles, edited subtitles, or no subtitles. All classes were conducted through an online class format via the video conferencing software, Zoom meetings. All students had to turn on their cameras while participating in the online classroom to let the instructor know whether learners were concentrating on the materials. The experimental procedure of this study is illustrated in Fig. 1, and the steps of the experiment are as follows: in the beginning step, all participants were required to do the TOEFL ITP listening comprehension test to classify and assign treatments. Next, the teacher opened the specific educational videos for each classroom based on the randomly assigned treatment (i.e., auto-subtitles, edited subtitles, and no subtitles). Then, after watching the videos, students completed the learning comprehension test of the videos’ story. It should be noted that the experiment with subtitle treatments ran for 2 weeks. Once students had watched the videos and completed the learning comprehension test, they were asked to complete the Cognitive Load and Learner Satisfaction scales. At the end of the experiment, all collected data were organised and processed for statistical analysis using IBM’s SPSS statistics software version 25.

## Results

### Effect on learning comprehension

The overall learning comprehension, before the adjustment of the mean with the analysis of covariate, is as follows, the mean score of all the participants (*M* = 4.81, *SD* = 1.96); auto-subtitles (*M* = 5.70, *SD* = 2.05); edited subtitles (*M* = 4.58, *SD* = 1.88); and the control group that had no subtitles (*M* = 4.12, *SD* = 1.63). Next, the mean scores for English listening comprehension were found to be all participants in the experiment (*M* = 10.15, *SD* = 3.49), auto-subtitles (*M* = 10.89, *SD* = 3.33), edited subtitles (*M* = 9.69, *SD* = 4.06), and no subtitles (*M* = 9.85, *SD* = 3.00).

A one-way ANCOVA was conducted to determine whether there was a statistically significant difference between auto-subtitles, edited subtitles, and no subtitles on the learning comprehension controlling for English listening comprehension. Lavene’s test indicated that the assumption of homogeneity of variance was not violated, (*F*_(2,76)_ = 0.31, *p* = 0.73). After controlling for English listening comprehension, there was a significant effect of condition on subtitle types, (*F*_(2,75)_ = 4.20, *p* = 0.02, *η*^*2*^ = 0.10) at 95% confidence interval (see Table 3). Estimated marginal means were significantly different from at least one method in the auto-subtitles (*M* = 5.56, *SE* = 0.34), edited subtitles (*M* = 4.67, *SE* = 0.34), and no subtitles (*M* = 4.18, *SE* = 0.34). Also, English listening comprehension was significantly related to subtitle types, (*F*_(2,75)_ = 11.32, *p* < 0.01, *η*^*2*^ = 0.13).

**Table 3:** The one-way ANCOVA result on overall learning comprehension controlling for English listening comprehension

Source	SS	df	MS	*F*	*η*^*2*^
Corrected model	70.21	3	23.40	7.63^*^	0.23
Intercept	64.93	1	64.93	21.18^*^	0.22
English listening comprehension	34.69	1	34.69	11.32^*^	0.13
Subtitle types	25.77	2	12.89	4.20^*^	0.10
Error	229.94	75	3.07		
Total	2128.00	79			
Corrected total	300.15	78			

*p < 0.05

**Fig. 2:**
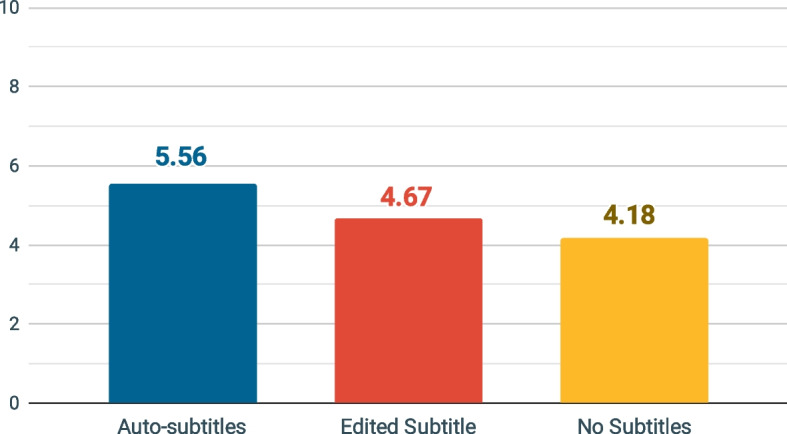
Estimated marginal means of overall learning comprehension controlling for English listening comprehension

Table 4 shows the results of a post hoc LSD test for multiple comparisons, which found that the mean value of learning comprehension was significantly different between auto-subtitles and no subtitles (*p* = 0.01, 95% CI = [0.42, 2.35]). However, there was no statistically significant difference in mean learning comprehension scores between auto-subtitles and edited subtitles (*p* = 0.07) or between edited subtitles and no subtitles (*p* = 0.32). Therefore, Fig. 2 is a chart that represents the estimated marginal means of learning comprehension among the three types of subtitles that control English listening comprehension: auto-subtitles (*M* = 5.56), edited subtitles (*M* = 4.67), and no subtitles (*M* = 4.18).

**Table 4:** Pairwise comparisons of learning comprehension

Test	MD	SE	*p*	95% CI	
				LB	UB
Auto-subtitles—Edited subtitles	0.90	0.49	0.07	− 0.07	1.86
Auto-subtitles—No subtitles	1.39	0.49	0.01	0.42	2.35
Edited subtitles—No subtitles	0.49	0.49	0.32	− 0.48	1.46

### Effect on cognitive load

This section examines the results of the cognitive load scale after watching the video with different assigned subtitles. The descriptive statistics of the overall cognitive load scale (7-level Likert scale) that was calculated by subtitle types; auto-subtitles (*M* = 4.33, *SD* = 0.66), edited subtitles (*M* = 4.20, *SD* = 0.60), and no subtitles (*M* = 4.14, *SD* = 0.69). Following this, a one-way ANOVA was performed to compare the effect of the three different subtitle types on the cognitive load scale. According to Table 5, the one-way ANOVA results revealed that there was not a statistically significant difference among the three groups (*F*_(2, 76)_ = 0.52, *p* = 0.60) at a 95% confidence interval.

**Table 5:** The one-way ANOVA results of cognitive load scale

Source	SS	df	MS	*F*
Between groups	0.50	2	0.25	0.52
Within groups	36.49	76	0.48	
Total	36.99	78		

### Effect on satisfaction

The last section represents the results of the learner satisfaction scale of using subtitles in educational videos with all different types of subtitles. The descriptive statistics of the overall learner satisfaction scale (4-level Likert scale) that is calculated by subtitle types; auto-subtitles (*M* = 2.75, *SD* = 0.63), edited subtitles (*M* = 2.69, *SD* = 0.60), and no subtitles (*M* = 2.99, *SD* = 0.52). Next, a one-way ANOVA was performed to compare the effect of the three different subtitle types on the learner satisfaction scale. According to Table 6, the one-way ANOVA result revealed that there was not a statistically significant difference among the three groups (*F*_(2, 76)_ = 2.00, *p* = 0.14) at a 95% confidence interval.

**Table 6:** The one-way ANOVA results of learner satisfaction scale

Source	SS	df	MS	*F*
Between groups	1.37	2	0.69	2.00
Within groups	26.04	76	0.34	
Total	27.42	78		

## Discussion

**RQ1: Can subtitles generated by an auto-subtitles system facilitate learning comprehension reach the same level as edited subtitles on an educational video?**

The results of the quantitative analysis showed that students using the auto-subtitles systems had higher mean scores for learning comprehension on how well they could remember the content of videos than those in the no-subtitles group. Additionally, there was no statistically significant difference between the auto-subtitles systems group and the edited subtitles group in mean video content-learning comprehension scores. As a result, the effects of subtitles in learning, which influence learning comprehension, can be explained by the appearance of virtual-text information and translation, which are described further below.

*Firstly*, the effects of using an auto-subtitles system appearing on educational videos can be explained by Mayer’s cognitive theory of multimedia learning. That is because a video’s visual–textual information positively affects learning captioning and can facilitate learning by helping students understand the content, improve concentration, and support disabled learners (Mayer, 2014). Furthermore, while watching an animated video, students mentioned they could listen to the audio information and read the linguistic information displayed on the screen simultaneously. This finding is similar to previous studies that emphasised how video captioning allows learners to realise the content because it promotes language learning by facilitating students to visualise what they hear (Danan, 2004). Therefore, subtitles in educational videos influence students’ cognitive process in understanding the content and facilitating learning more than just listening to the audio.

Additionally, student number 60 commented on the use of subtitles in educational videos by suggesting, “*Subtitles make viewers understand content easier and faster. The subtitles with keywords in videos can help with taking notes in learning.*” This opinion is consistent with previous studies’ assumption that the videos’ keyword-focused subtitles will effectively enhance student learning (Cao et al., 2019; Hsieh, 2019). Thus, subtitles with keywords will help to emphasise the video’s message in their verbal language by allowing learners to grasp the concept from the videos.

*Secondly*, the reliance on translation is related to language proficiency. The outcomes revealed a positive relationship between language proficiency (listening comprehension) and learning comprehension of video content with and without subtitles. This correlation is consistent with student perceptions of language proficiency level, which refers to language proficiency as a barrier to learning through video content in a foreign language that students feel uncomfortable or unable to communicate. Foreign language proficiency problems affecting learning through videos are consistent with the previous study by Nonthamand (2017). Also, learners with high language skills are more likely to understand video content than those with low language skills (van der Zee et al., 2017). According to the learning satisfaction questionnaires, students in the group without subtitles stated that it was too difficult to listen to and understand the information since they were not good at English. While students in the edited-subtitles group believed that subtitles help non-native English speakers understand the video’s content. Therefore, subtitles are especially helpful for learners with low language skills or who need tools to understand a non-first language. This finding is consistent with the bilingual dual-coding theory (Paivio & Desrochers, 1980), which asserts that humans have a good memory when they receive information from more than two channels (i.e., verbal or auditory information and image or visual information).

Furthermore, subtitles in L1 might assist students in improving their learning of the L2. They commented on the benefits of utilising their L1 in their learning and practising English as their L2 in this study. From the open-ended questions, students reflected on watching videos with subtitles to learn others’ content in English. They also believe they can learn other subjects while practising English listening and learning new vocabulary. Therefore, when utilising subtitles in an educational video in English as an L2 course with native Thai-speaking learners, the students realise the benefits of using subtitles in their learning of foreign languages. These comments are consistent with Danan’s (2004) conclusion that subtitles play a critical role in translating verbal information in L1 into L2, increasing language comprehension, and leading to additional cognitive benefits, such as greater depth of processing. Added to this is the assumption of Guillory (1998) that reading subtitles are an easily performed act that makes learners feel comfortable. Additionally, the message translated from machine translation facilitates students’ vocabulary learning (Fuji, 1999; Hsieh, 2019; Lee, 2019; Niño, 2009, 2020). As a result, the Thai subtitle from the automatic subtitle system, which was conducted in this study, could improve participants’ comprehension of video content in English in computing science subjects.

**RQ2: Do auto-subtitles and edited subtitles have different levels of cognitive load?**

According to the results of this study, there was no significant difference among the three types of subtitles on levels of cognitive load. More specifically, this project’s results matched Chan et al.’s (2020) as they both found that auto-generated captions and correctly edited captions have no significant effect on cognitive load. The causes behind these results will be discussed in this section. Regarding the results, the four following issues could demonstrate why no significant difference was found between the experimental groups and the control group:

*First*, edited subtitles in Thai may be wordy and complex due to the translation process affecting the learner’s efforts to understand. A student in the edited subtitles group gave their opinions on the use of educational videos with subtitles as follows:

I want you to be careful about the translated subtitles because some parts of them can’t convey the concepts. There are too many translated words into Thai making it difficult to read. Maybe transliteration words are easier to communicate than translating into Thai. (Student number 41)

The student’s feedback demonstrates an attempt to comprehend content by matching the narrative in English with the Thai subtitles that appear. The cognitive load arising from the simultaneous encoding of information in two languages is consistent with the theory of bilingual dual coding by Paivio and Desrochers (1980). These connections between the L1 and L2 occur between translation-equivalent logogens. This interaction could be regarded as a subset of verbal associative connections with a high likelihood of activation in code-switching tasks (Paivio, 2014). Consequently, students may handle a cognitive load while encoding in bilingual and simultaneously interpreting differences in language structures when reading subtitles in Thai.

*Second*, the requirement for subtitles may vary depending on learners’ English proficiency. Since the participants had a high score on the English listening comprehension test (*M* = 10.15, *SD* = 3.49), learners in this study may have relied on their English language skills to interpret the video content rather than using Thai subtitles. This finding is reflected in the opinions of one participant who reported total English language proficiency scores and stated, *“Listening to audio is easier than reading subtitles to understand content. Also, subtitles in English may be better than subtitles in Thai”* (Student number 47). This opinion demonstrates that students with high English language skills may not rely on Thai subtitles when watching videos in English. This result is similar to Niño’s (2020) study, which found that students with higher language proficiency were more aware of the inaccuracy and claimed that they do not require the translation output from online machine translations for comprehension purposes. Moreover, this finding again relates to the bilingual dual coding theory (Paivio, 2014) because when students have a high level of bilingual proficiency, they may select whichever language option minimises the cognitive load that would be incurred by dealing with simultaneous bilingual inputs. In consequence, this could be assumed that the students in the experimental subtitling group may not have given the weight to understand the video content by reading the subtitles.

*Third*, students may suffer from cognitive overload and be unable to decode all information while experiencing cognitive overload due to watching videos containing too much verbal and visual information (Sweller et al., 1998). In this experiment, students using editorial subtitles reported that sometimes it is difficult to concentrate while reading subtitles and that low concentration is an obstacle to learning the video’s content. Nevertheless, there is a difference in the appearance of video subtitles on the YouTube platform, with automated subtitles being synchronous word-by-word, whereas editorial subtitles are whole sentences. Due to this difference in the formation of subtitles, learners may concentrate more on the gradually emerging stimuli and less on the content. This result is consistent with Shadiev et al. (2015)’s learning concentration study in an auto-caption system that allows learners to track the content being lectured and Kruger et al. (2017)’s study, which found that subtitles increase immersion with characters that fulfil a focusing effect to gain a stronger connection with the story.

*Fourth*, students may already have background knowledge of the topic being studied. At the end of the learning comprehension assessment, students can review their grades and the correct answers.

In one case, a student in the auto-subtitles group took the time to inquire about an incorrect test answer in the first video after the class ended. During the exchange, the student argued that his answer should be correct based on his prior knowledge of the topic. This discussion is noteworthy because the learner’s prior understanding of the additional content was not presented in the lecture video. This behaviour suggests that in experiments conducted, students may use prior knowledge to learn rather than learning new information contained in video learning activities.

As a consequence of the notions mentioned above, it was determined that there was no statistically significant difference in the level of cognitive load found between the different Thai subtitle formats (i.e., auto-subtitles, edited subtitles, and no subtitles) in this sample of computer science classrooms using educational videos in English.

**RQ3: Are students satisfied with using auto-subtitles systems?**

According to this study’s results, students in the auto-subtitling group were more satisfied with using Thai subtitles in educational videos in English. Providing subtitles in Thai as a first language makes students feel more comfortable because they can readily access meaning while watching foreign-language media (Guillory, 1998). In addition, the participants in the auto-subtitles group highlighted the advantages of utilising subtitles to learn through educational videos and that they are delighted to use the auto-subtitle system for watching others’ videos in foreign languages. They also mentioned that they could activate foreign language video clips they want to study for better understanding. These opinions demonstrate that the auto-subtitles system in the Thai language is beneficial to their learning, such as taking notes from the subtitles translated into Thai language or implementing the auto-subtitles system to translate other videos from foreign languages. Reading the subtitles while listening to and watching the video is also helpful in recapitulating the content (Liyanagunawardena, 2021). Therefore, using automated subtitles in educational videos in English is beneficial for improving positive learning attitudes among the sample participants.

### Practical implications

This study found that the Thai subtitle generated by the auto-subtitles system in English educational videos could help students who require the translation subtitles to understand the video’s content in a language they are comfortable with. Although the quality of translated subtitles from the auto-subtitles system may be a concern for implementation in some languages (Niño, 2009; Briggs, 2018), these experiments with Thai subtitles generated from the auto-subtitles system in educational videos in English could be used in comparison with the study of using the same system of translation from English into Thai (Wongprom & Thitthongkam, 2018; Yuchareon, 2017). However, before using an automatic subtitle system in the classroom, instructors should ensure that the generated subtitles have been translated accurately.

### Limitations

This study had limitations related to the sample population. During the Covid-19 pandemic recruiting samples from schools ready for online learning and experimentation learning activities is a concern. In particular, it must include the requirements of schools and participants equipped with devices and network connections. As a result, the experiment of the sample group was limited to only one school in Thailand’s capital city, Bangkok. The sample group contained students with high background knowledge and advanced English skills due to social context. Moreover, the sample size was small, and the experimental period was limited. Plus, it was necessary to remove some students’ records who did not participate in both trials due to the students’ leaving the class for vaccination on the day of the experiment.

Furthermore, because this study experiment applies a Posttest-Only Control Group Design, it is difficult to discern the student’s prior knowledge of the video content, which could affect their learning comprehension and cognitive load. As a result, having a pre-study test is one method that could assist in controlling the experiment’s results more precisely.

Lastly, the instruments used in this study were adapted from previously reviewed studies on translating English to Thai instead of creating new instruments specifically in the Thai language. Also, due to the limited time and number of schools for this experiment, there was no opportunity to pilot the instruments before collecting data. Therefore, there may be restrictions on references to population groups due to the limitations mentioned above.

### Suggestions for future study

From this study, there are related issues arising from observations during the experimental as follows:

*Firstly*, the videos used in this study differed in presentation. The first video in the experiment was a geometric motion graphic with no virtual graphics. On the other hand, the second video had redundancy animation, which is a visual representation of a machine’s algorithm that includes keyword text as cognitive cues. Interestingly, the results of the video learning comprehension scores following the first video revealed that students who watched with and without subtitles performed statistically differently. However, there was no statistically significant difference in the learning comprehension scores after the second video.

*Secondly*, this experiment should be replicated with a larger sample, hierarchically dispersed model with a wide variety of language ability levels for the results to be referenced to a population. Additionally, standard language proficiency levels, such as the Common European Framework of Reference for Languages (CEFR), may be classified in future studies to distinguish results based on the learner’s level of language proficiency. In addition, experiments should include a pre-study test of students’ prior knowledge related to learning content.

*Lastly*, at the time of this research experiment, there was no automatic subtitle system in the videos that could translate Thai into other languages. As a result, studying and developing an automated subtitle system that supports Thai translation in videos will greatly benefit open education broadly.

Consequently, for future research on the implementation of subtitle system tools in educational videos, the recommendations above should be considered to resolve any weaknesses and be used as caution in future studies.

## Conclusion

This study aimed to examine the effects of three different types of subtitles in the Thai language (i.e., auto-subtitles, edited subtitles, and no subtitles) on learning comprehension, cognitive load, and satisfaction to determine whether it is feasible to use AI technology as an auto-subtitles system to facilitate online learning with educational videos. Based on the quantitative analysis and qualitative interpretation of this study, it was concluded that the auto-subtitles system that generated Thai language subtitles for English educational video facilitated higher learning comprehension, lessened cognitive load, and had an increased degree of satisfaction compared to editorial subtitles created by Thai natives.

These results indicate that Thai subtitles generated by the auto-subtitles system in English language educational videos can facilitate students’ learning comprehension, cognitive load, and satisfaction in using the system.

## Data Availability

Not applicable.

## Abbreviations

ASR: Automatic Speech Recognition
L1: First language
L2: Second language
NMT: Neural Machine Translation
TOEFL ITP: Test of English as a Foreign Language Institutional Testing Program
